# Monitoring circulating tumor DNA revealed dynamic changes in *KRAS* status in patients with metastatic colorectal cancer

**DOI:** 10.18632/oncotarget.25309

**Published:** 2018-05-11

**Authors:** Yuji Takayama, Koichi Suzuki, Yuta Muto, Kosuke Ichida, Taro Fukui, Nao Kakizawa, Hideki Ishikawa, Fumiaki Watanabe, Fumi Hasegawa, Masaaki Saito, Shingo Tsujinaka, Kazushige Futsuhara, Yasuyuki Miyakura, Hiroshi Noda, Fumio Konishi, Toshiki Rikiyama

**Affiliations:** ^1^ Department of Surgery, Saitama Medical Center, Jichi Medical University, Omiya-ku, Saitama 330-8503, Japan; ^2^ Department of Surgery, Nerima-Hikarigaoka Hospital, Nerima-ku, Tokyo 179-0072, Japan

**Keywords:** liquid biopsy, circulating tumor DNA, colorectal cancer, KRAS, droplet digital PCR

## Abstract

*KRAS* mutated circulating tumor DNA (MctDNA) can be monitored in the blood of patients with metastatic colorectal cancer (mCRC), but dynamic changes have not been determined. Four hundred and fifty-seven plasma samples were collected prospectively from 85 mCRC patients who underwent chemotherapy. MctDNA in plasma was detected by droplet digital PCR, and the percentage of MctDNA in total circulating cell-free DNA was calculated. *KRAS* assessment in tumor tissues showed 29 patients with the mutant-type (MT) and 56 patients with the wild-type (WT). Twenty-three of 29 MT patients (79.3%) and 28 of 56 WT patients (50.0%) showed MctDNA. Emergence of MctDNA was recognized during treatments with various drugs. Regardless of *KRAS* status in tumor tissues, patients with MctDNA in blood showed poor progression-free survival with first-line treatment. Median percentage of MctDNA accounted for 10.10% in MT patients and 0.22% in WT patients. These differences between MT and WT likely affected patterns of changes in MctDNA. *KRAS* monitoring identified dynamic changes in MctDNA, such as continuous, intermittent, and transient changes (quick elevation and disappearance). Emergence of MctDNA involved drug resistance, except for transient changes, which were seen in WT patients and likely corresponded with the drug response. Transient changes could be involved in recovery of sensitivity to anti-EGFR antibody in WT patients. Monitoring MctDNA during various treatments showed dynamic changes in *KRAS* status and could provide useful information for determining treatments for patients with mCRC.

## INTRODUCTION

Genotyping of oncogenic *RAS* mutations is routinely undertaken as it is an important biomarker used to predict drug resistance to epidermal growth factor receptor (EGFR)-targeted monoclonal antibodies in patients with metastatic colorectal cancer (mCRC) [1, 2, 3]. In this approach, tumor tissues are used to explore representative genomic profiles of the tumor. However, discrepancies in the genomic profile can occur because of the heterogeneous nature of a tumor (intratumor heterogeneity) [4–7]. Differences in genomic profiles between primary tumors and distant metastases have also been reported in 10% of mCRC [4]. The genomic profile of the tumor, which is representative of the tumor molecular landscape, can be altered during chemotherapy with commonly used cytotoxic agents [8] as well as targeted drugs [9–12]. Because of the possible implications of these factors on the molecular profile, tumor tissue-based genotyping has some limitations in attempts to identify the molecular features of the tumor.

A blood-based technology platform that tracks circulating tumor DNA (ctDNA), known as liquid biopsy, could be an ideal alternative to a biopsy of tumor tissue [13], and may remove the restrictions associated with the use of tissue samples [14]. This technique reflects tumor dynamics [15] and allows multiple testing over time, monitoring real-time changes within the tumor and evaluation of therapeutic responses [9–11, 16–19, 20]. BEAMing technology and digital PCR, one of the platforms of the liquid biopsy using micro-compartmentalization of PCR, can detect rare mutant alleles in blood with a high sensitivity of 0.01 to 0.001% [21, 22]. These blood-based platforms with their high sensitivity enable monitoring of tumor dynamics by tracking ctDNA during treatment in patients with mCRC [15]. Tumor dynamics obtained from *KRAS* monitoring could provide important information about treatment strategies for patients with mCRC, such as detection of drug resistance to anti-EGFR antibody before radiographic documentation of disease progression [10, 9, 19]. Additionally, they raise the possibility of an alternative molecular explanation for the efficacy of re-challenge therapies based on EGFR blockade [19].

Despite the clinical advantages obtained by tracking *KRAS* mutated ctDNA (MctDNA), the dynamics of MctDNA during regimens currently in use in clinical practice are not well known in patients with mCRC. Details and the clinical significance are important to help determine the best anti-cancer treatment as a precision medicine. Further exploration is required for clinical application. In this study, we examined the dynamics of MctDNA during various regimens for mCRC and determined the characteristics and clinical significance of the method.

## RESULTS

### Assessment of KRAS mutations in blood and tissue

**Figure 1:**
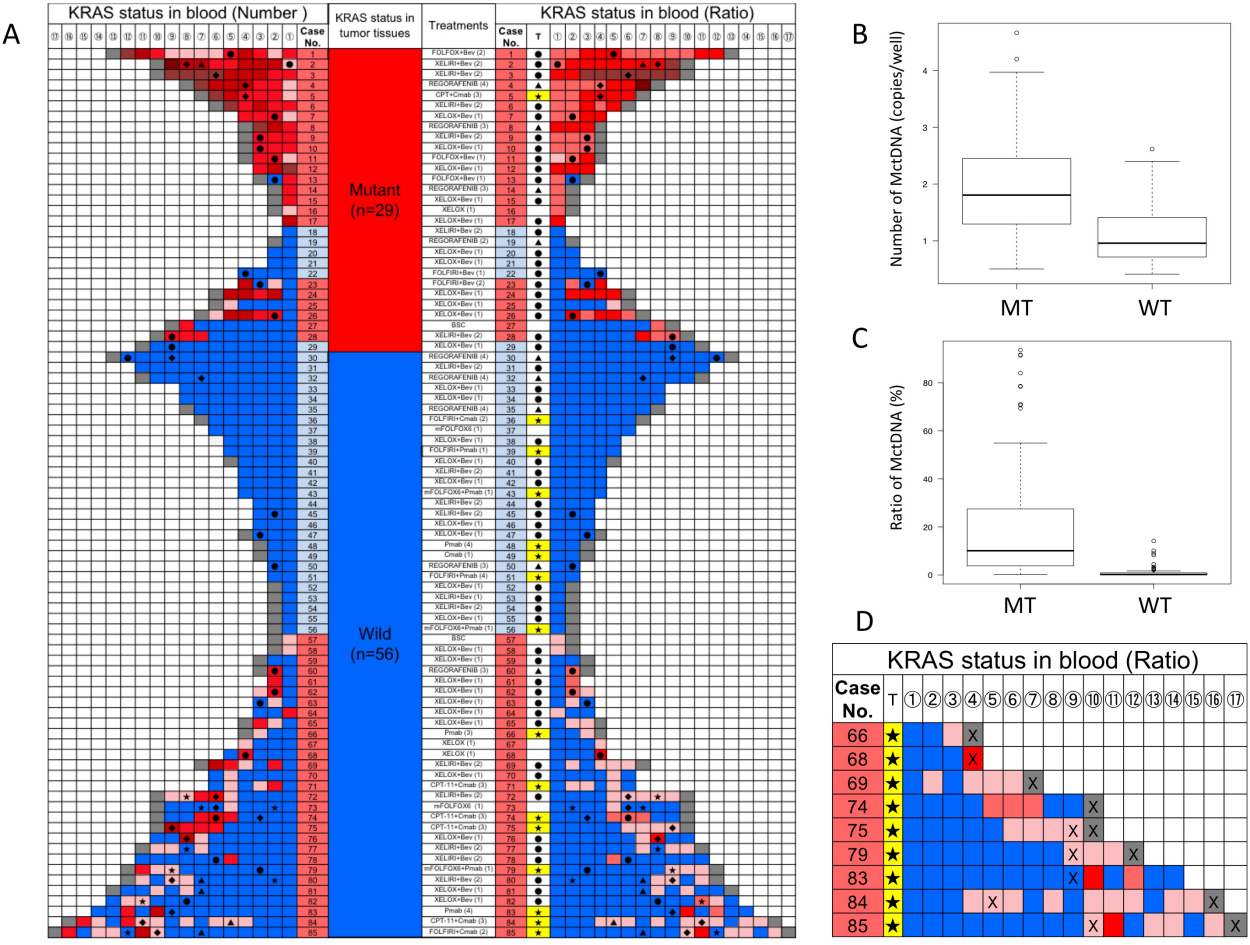
*KRAS* monitoring of mCRC patients and comparison of MctDNA between MT and WT **(A)** *KRAS* monitoring of mCRC patients treated with various drugs across several treatment lines. Initial assessments for circulating tumor DNA with *KRAS* mutations (MctDNA) varied by treatment line and regimen and are shown under “treatment (lines)”; (XELOX (1) means that XELOX was given as the first-line treatment). *KRAS* status in tumor tissues is shown. Patients with mutations (red), those without (blue). *KRAS* assessment in tumor tissues are under “*KRAS* status in tumor tissues” with red for patients with the mutant-type (MT) and blue for patients with the wild-type (WT). Monitoring MctDNA is shown under “*KRAS* status in blood”, ordered by timing of blood examination (①→⑯). MctDNA was assessed using two methods for “*KRAS* status in blood”. Left column under “*KRAS* status in blood (number)” indicates the number of MctDNA. MctDNA not detected (blue); detection of MctDNA in fewer than 10 copies/well (pink); 10 ≤ MctDNA < 50 copies/well (light red); 50 ≤ MctDNA < 100 copies/well (red); 100 ≤ MctDNA < 100 copies/well (light brown); MctDNA ≥ 1000 copies/well (brown); end of treatment because of disease progression (gray). Right column under “*KRAS* status in blood (ratio)” shows ratio of MctDNA among total circulating cell-free DNA (MctDNA and circulating cell-free DNA without *KRAS* mutations). The mutation ratio was calculated by fractional abundance (MctDNA/ total circulating cell-free DNA). MctDNA not detected (blue); detection of MctDNA less than 1% (pink). MctDNA ≤ 1 < 10% (light red); 10 ≤ MctDNA < 30% (red); 30 ≤ MctDNA < 50% (light brown); MctDNA ≤ 100% (brown); end of treatment because of disease progression (gray). ●: anti-VEGF antibody; ▲: regorafenib; ★: anti-EGFR antibody; ◆: TAS-102. **(B)** Comparison of number of MctDNA between patients with MT and WT in tumor tissues. Vertical axis represents logarithm. **(C)** Comparison of ratio of MctDNA between patients with MT and WT in tumor tissues. The mutation ratio was calculated by fractional abundance (MctDNA / total circulating cell-free DNA). Vertical axis represents mutation ratio × 10^2^. **(D)** Initial detection of MctDNA in patients with WT treated with anti-EGFR antibody. T: treatment; ★: anti-EGFR antibody; X: detection of radiological disease progression. Detection of MctDNA less than 1% (pink); MctDNA ≤ 1 < 10% (light red); 10 ≤ MctDNA < 30% (red).

A *KRAS* monitoring image from mCRC patients treated with various drugs during the treatment lines is shown in Figure 1A. *KRAS* assessment in tumor tissues identified 29 patients with the mutant-type (MT) and 56 patients with the wild-type (WT). Assessment of *KRAS* status in blood incorporated both the number of MctDNA and the ratio of MctDNA.

### Dynamics of KRAS mutated circulating tumor DNA and its impact on outcome in patients with the mutant-type

**Figure 2:**
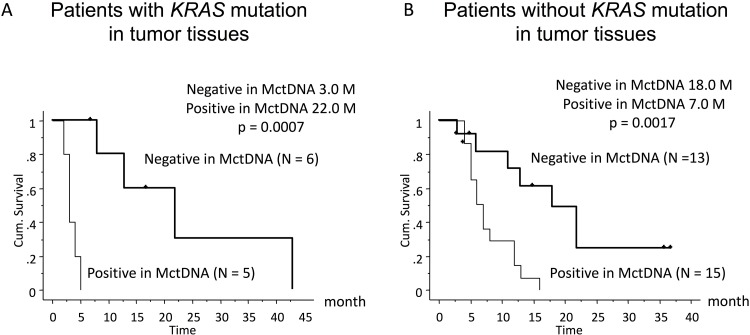
Comparison of progression-free survival (PFS) in patients treated with the first-line therapy according to *KRAS* status in blood Patients with *KRAS* mutations in tumor tissues (left) and those without *KRAS* mutations (right).

**Figure 3:**
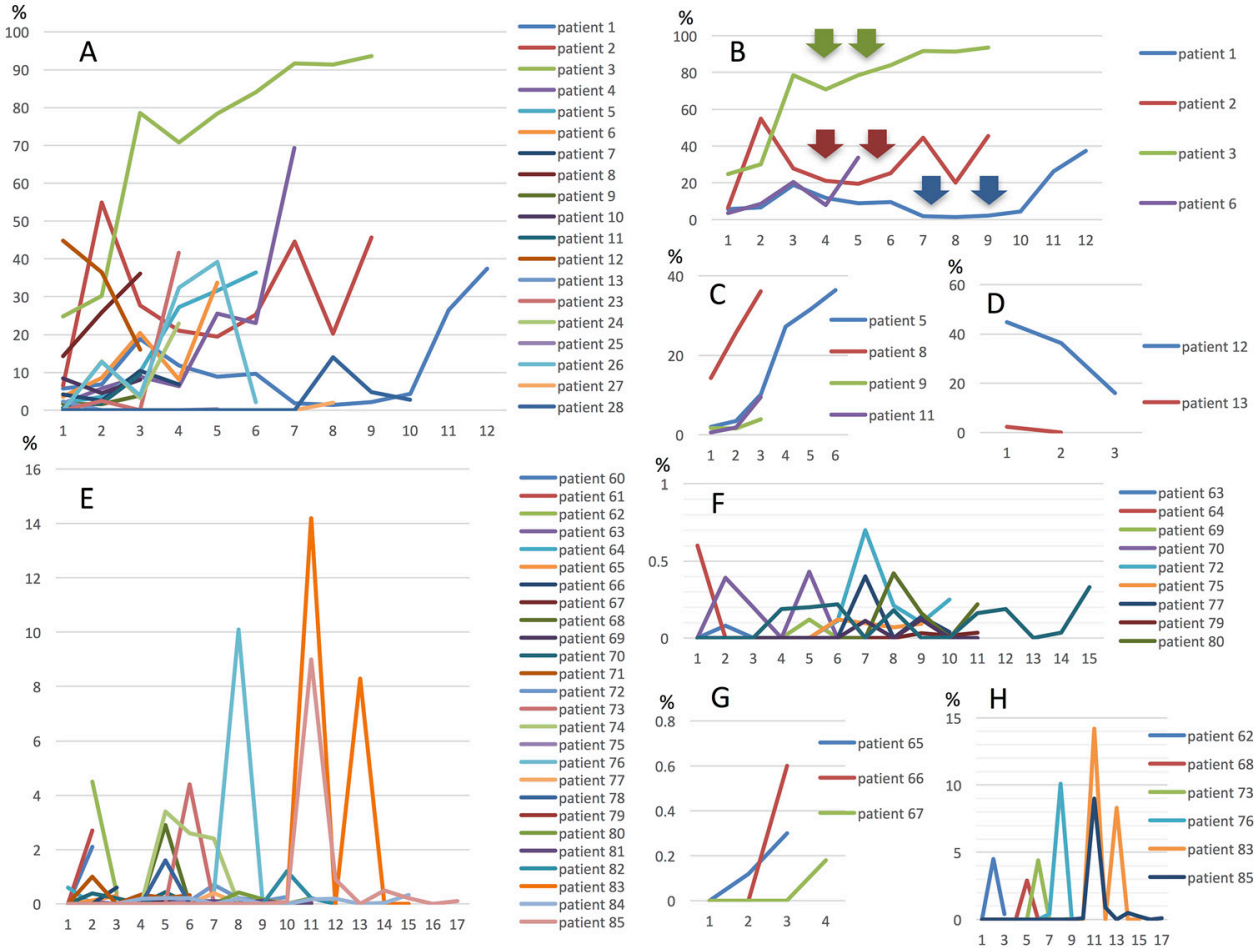
Changes in MctDNA during treatment in 29 patients with mutations in tumor tissues **(A)**, representative graph of four patients with stable levels of MctDNA **(B)**, four patients with increases **(C)**, and two patients with decreases **(D)**. Changes in MctDNA during treatment in 28 patients without mutations in tumor tissues **(E)**, representative graph of nine patients with intermittent changes in MctDNA **(F)**, three patients with increases **(G)**, and seven patients with a spike in elevation **(H)**. Y axis shows ratio of MctDNA (%) and X axis shows timing of blood examination.

**Figure 4:**
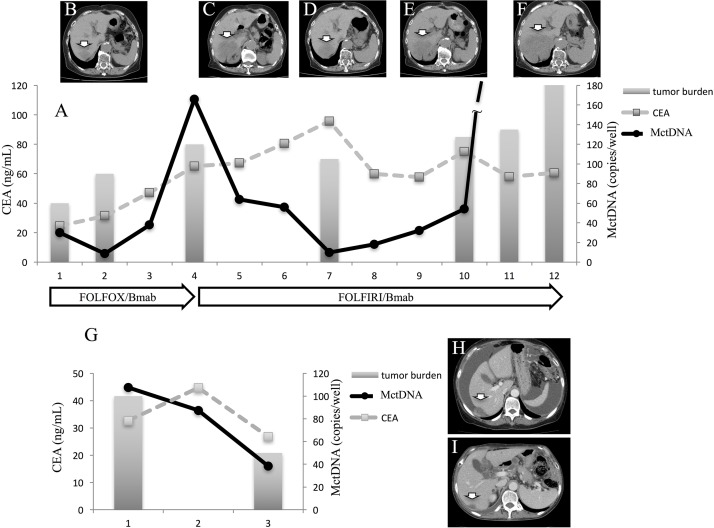
Clinical course of a mCRC patient with multiple liver metastases with a long stable disease **(A** and **G)** and computed tomography (CT) **(B–F)**. A 75-year-old woman with multiple liver metastases is denoted as patient 1. She was treated with FOLFOX + bevacizumab for the first-line treatment. (A) and (B) show CT images before and after treatment with FOLFOX + bevacizumab. Increased levels of MctDNA were observed before radiological progression (B). FOLFIRI + bevacizumab was administrated as the second-line treatment. The tumor did not change in size (C, D, and E) and levels of MctDNA were stable for a time with the second-line treatment. Progression-free survival of 6 months was achieved with stable levels of MctDNA (A), followed by progression detected by CT (F). A 66-year-old woman with multiple liver metastases and ascites is denoted as patient 12. She was treated with XELOX + bevacizumab for the first-line treatment. **(H)** and **(I)** show CT images before and after treatment with XELOX + bevacizumab. The tumor shrank and levels of MctDNA decreased (I). Arrow shows liver metastasis. CEA: carcinoembryonic antigen.

In 29 patients with the MT, MctDNA was detected in 23 patients (79.3%) (Figure 1A). Among 18 patients who underwent second-line or subsequent treatment lines, MctDNA was detected in 17 patients (94.4%). Details of the clinical course of these 29 patients is shown in Table 1. The median value of MctDNA was 64.0 copies/well (3.2–45800) for the number and 10.10% (0.26–93.60) for the ratio in these 29 patients (Figure 1B and 1C). Comparing progression-free survival (PFS) of the first-line treatment between patients with MctDNA and without, there was a significant difference in PFS (Figure 2A), with a worse outcome in patients with MctDNA (22.0 vs 3.0 months, p = 0.0007). Most patients showed a continuous change with increasing MctDNA (Figure 3A and 3C). Four patients had a stable MctDNA level, suggesting a long stable disease (Figures 3B and 4A), and two patients showed a quick decrease in MctDNA with shrinkage of tumors in response to treatment (Figure 3D and Figure 4G).

**Table 1:** Clinical information for patients with the mutant-type

Case	Sex	Age	Primary site	Metastatic site	*KRAS* primary tissue	*KRAS* MctDNA	1st line	2nd line	3rd line	4th line	5th line
1	f	78	S/C	Liver, lung	G12S	G12S	mFOLFOX6/Bev	FOLFIRI/Bev	BSC		
2	m	49	Rectum	Liver	G12V	G12V	XELOX/Bev	XELIRI/Bev	Regorafenib	Lonsurf	BSC
3	f	73	T/C	Liver	G12C	G12C	XELOX/Bev	XELIRI/Bev	Lonsurf	BSC	
4	m	69	A/C	Liver	G13D	G13D	FOLFIRI	CPT-11/Cmab	Regorafenib	Lonsurf	BSC
5	m	80	Rectum	Lung, LN	G13D	G13D	XELOX	XELIRI	CPT-11/Cmab	Lonsurf	BSC
6	f	67	Rectum	Liver, lung, LN	G12D	G12D	XELOX/Bev	XELIRI/Bev	Regorafenib	Lonsurf	BSC
7	m	19	S/C	LN	G12D	G12D	XELIRI/Bev	XELOX/Bev			
8	m	52	A/C	Liver	G12D	G12D	XELOX/Bev	XELIRI/Bev	Regorafenib	BSC	
9	f	67	A/C, S/C	LN	G12C	G12C	XELOX/Bev	XELIRI/Bev	Lonsurf	BSC	
10	m	78	A/C	Liver	G13D	G13D	XELOX/Bev	XELIRI/Bev	BSC		
11	m	65	A/C	Lung	G12C	G12C	mFOLFOX6	FOLFIRI/Bev	Lonsurf	BSC	
12	f	66	A/C	Peritoneum	G12S	G12S	XELOX/Bev				
13	m	79	A/C	Liver, peritoneum	G13D	G13D	mFOLFOX6/Bev	FOLFIRI/Bev	Lonsurf	Regorafenib	BSC
14	f	76	Rectum	Liver, peritoneum	G12D	G12D	XELOX/Bev	XELIRI	Regorafenib	BSC	
15	m	76	Rectum	Liver, LN	G12D	G12D	XELOX/Bev	XELIRI/Bev	Regorafenib	Lonsurf	BSC
16	f	66	Rectum	Lung, LN	G12S	G12S	XELOX	XELIRI	BSC		
17	m	71	T/C	Liver, lung, peritoneum	G12D	G12D	XELOX/Bev	Lonsurf/Bev			
18	m	59	Rectum	Lung	G12V	N.D.	XELOX/Bev	XELIRI/Bev			
19	m	72	A/C, D/C	Liver, peritoneum	G12C	N.D.	FOLFIRI/Bev	Regorafenib	BSC		
20	f	82	Rectum	Liver, lung	G12D	N.D.	XELOX/Bev				
21	f	73	S/C	Liver, peritoneum	G12V	N.D.	XELOX/Bev				
22	m	76	A/C	Peritoneum, LN	G12C	N.D.	mFOLFOX6	FOLFIRI/Bev	Regorafenib		
23	f	71	Cecum	Peritoneum	G12A	G12A	mFOLFOX6	FOLFIRI/Bev	Lonsurf		
24	f	68	A/C	Liver, lung	G12D	G12D	XELOX/Bev	Pmab	BSC		
25	m	62	Rectum	Liver, LN	G12D	G12D	XELOX/Bev	BSC			
26	m	33	Rectum	Liver, peritoneum	G12D	G12D	XELOX/Bev	XELIRI/Bev	BSC		
27	m	67	Rectum	Lung	G12V	G12V	BSC				
28	f	61	Rectum	Lung	G12C	G12C	XELOX/Bev	XELIRI/Bev	Regorafenib	BSC	
29	m	50	Rectum	Liver, LN	G12V	N.D.	XELOX/Bev	XELIRI/Bev	Regorafenib	Lonsurf	BSC

A/C: ascending colon; T/C: transverse colon; D/C: descending colon; S/C: sigmoid colon; LN: lymph node; mFOLFOX6: oxaliplatin, folinic acid, and fluorouracil; FOLFIRI: irinotecan, folinic acid, and fluorouracil; XELOX: capecitabine and oxaliplatin; XELIRI: capecitabine and irinotecan; Bev: bevacizumab; Pmab: panitumumab; Cmab: cetuximab; CPT-11: irinotecan; BSC: best supportive care.

### Dynamics of mutated circulating tumor DNA and outcome in patients with the wild-type

In 56 patients with the WT, MctDNA was detected in 28 patients (50.0%) (Figure 1A). Details of the clinical course of these 56 patients is shown in Table 2. MctDNA was detected in patients treated with anti-EGFR antibody as well as those treated with other drugs such as anti-vascular endothelial growth factor (VEGF) antibody, regorafenib, and TAS-102. Cytotoxic agents without targeted therapies also showed the emergence of MctDNA (Table 3). The median value of MctDNA was 9.1 (2.6–414) for the number and 0.22% (0.002–14.2) for the ratio (Figure 1B and 1C). The median value of MctDNA for the number detected in patients with the WT was 1/7 of that in patients with the MT, suggesting that about 1/7 of tumor cells (14.7%) in patients with the WT might have the *KRAS* mutation. Comparing first-line treatment between patients with MctDNA and those without, there was a significant difference in PFS (Figure 2B), showing a worse outcome in patients with MctDNA (18.0 vs 7.0 months, p = 0.0017). Most patients showed intermittent changes in elevation; with low levels of MctDNA generally (Figure 3E and 3F), four patients showed a continuous change with an increase (Figure 3G), and five patients showed a transient change with a spike in elevation (quick elevation and disappearance) (Figure 3H), with close to 10 times the amount of MctDNA as the intermittent change in elevation (Figure 3F). Initial detection of MctDNA in WT patients treated with anti-EGFR antibody was likely prior to radiological disease progression (Figure 1D).

**Table 2:** Clinical information of patients with the wild-type

Case	Sex	Age	Primary site	Metastatic site	*KRAS* Primary tumor	*KRAS* MctDNA	1st line	2nd line	3rd line	4th line	5th line	6th line	7th line
30	m	69	Rectum	LN	WILD	N.D.	XELOX/Bev	XELOX	Pmab	Regorafenib	Lonsurf	Pmab	BSC
31	m	64	S/C	Liver	WILD	N.D.	XELOX	XELIRI/Bev					
32	m	70	Rectum	Lung, LN	WILD	N.D.	XELOX	XELIRI/Bev	CPT-11/Cmab	Regorafenib	Lonsurf	BSC	
33	m	58	Rectum	Liver, peritoneum	WILD	N.D.	XELOX/Bev						
34	m	74	S/C	Lung, LN	WILD	N.D.	XELOX/Bev						
35	m	74	Cecum	Liver, lung, LN	WILD	N.D.	XELOX/Bev	XELIRI/Bev	CPT-11/Cmab	Regorafenib	Lonsurf	BSC	
36	m	68	S/C	Peritoneum	WILD	N.D.	FOLFIRI/Cmab	BSC					
37	f	47	Rectum	Liver	WILD	N.D.	mFOLFOX6/Bev						
38	f	75	S/C	Peritoneum	WILD	N.D.	XELOX/Bev						
39	f	74	S/C	Peritoneum	WILD	N.D.	FOLFIRI/Pmab						
40	m	47	A/C	Liver, LN	WILD	N.D.	XELOX/Bev	BSC					
41	m	72	Rectum	Liver	WILD	N.D.	XELIRI/Bev						
42	m	52	T/C, Rectum	Liver	WILD	N.D.	XELOX/Bev						
43	f	47	Rectum	Liver, LN	WILD	N.D.	mFOLFOX6/Pmab						
44	m	74	Cecum	Liver	WILD	N.D.	XELOX	XELIRI/Bev					
45	m	78	Rectum	LN	WILD	N.D.	XELOX/Bev	XELIRI/Bev	CPT-11/Pmab				
46	f	55	S/C	Liver	WILD	N.D.	XELOX/Bev	XELIRI/Bev					
47	f	70	D/C	Peritoneum	WILD	N.D.	FOLFIRI/Bev	mFOLFOX6/Bev	CPT-11/Cmab	Regorafenib	BSC		
48	m	58	Rectum	Liver	WILD	N.D.	mFOLFOX6/Bev	FOLFIRI/Bev	Pmab	BSC			
49	f	75	A/C	Liver	WILD	N.D.	Cmab	BSC					
50	f	68	A/C	Peritoneum	WILD	N.D.	mFOLFOX6/Bev	FOLFIRI/Pmab	Lonsurf	Regorafenib			
51	m	70	S/C	LN	WILD	N.D.	XELOX/Bev						
52	m	70	S/C	Bone, LN	WILD	N.D.	XELOX	BSC					
53	m	72	A/C, Rectum	Liver, lung	WILD	N.D.	XELOX/Bev	XELIRI/Bev	CPT-11/Cmab	Regorafenib	Lonsurf	BSC	
54	m	67	Cecum	Peritoneum	WILD	N.D.	XELOX/Bev	FOLFIRI/Pmab	BSC				
55	f	57	Rectum	Liver, peritoneum	WILD	N.D.	mFOLFOX6/Pmab	BSC					
56	f	66	S/C	Liver, lung	WILD	N.D.	BSC						
57	m	70	A/C	Liver, peritoneum	WILD	N.D.	XELOX/Bev	XELIRI/Bev	BSC				
58	f	62	S/C	Peritoneum	WILD	12V	XELOX/Bev	XELIRI/Bev	Regorafenib	BSC			
59	f	75	Rectum	Liver	WILD	12V	XELOX	FOLFIRI/Pmab	Regorafenib	BSC			
60	m	52	S/C	Liver, lung	WILD	12D	XELIRI/Bev	FOLFIRI/Cmab	BSC				
61	f	47	Rectum	Liver	WILD	12C, 12R	XELOX/Bev						
62	f	69	Rectum	Liver, LN	WILD	12S	XELOX/Bev	XELIRI/Bev					
63	f	72	T/C	Liver	WILD	12D	XELOX/Bev	XELIRI/Bev	Lonsurf/Bev	Regorafenib			
64	m	74	Rectum	Lung	WILD	12R	XELOX/Bev	XELIRI/Bev	CPT-11/Pmab				
65	m	61	S/C	Liver	WILD	12C, 13D	XELOX/Bev	BSC					
66	m	53	A/C	Peritoneum	WILD	12D	XELOX/Bev	XELIRI/Bev	Pmab	BSC			
67	f	67	S/C	Locally advanced	WILD	12S	XELOX						
68	m	65	T/C	Peritoneum	WILD	12S	FOLFIRI/Bev	FOLFIRI/Pmab					
69	m	49	S/C	Liver, peritoneum	WILD	12A, 12C, 12S	XELOX/Bev	XELIRI/Bev	CPT-11/Cmab	Lonsurf	BSC		
70	f	64	Rectum	Liver, LN	WILD	13D	XELIRI/Bev	Lonsurf/Bev					
71	f	79	Rectum	LN	WILD	12D, 13D	XELOX/Bev	XELIRI/Bev	CPT-11/Cmab				
72	m	57	Rectum	Liver	WILD	12V	SOX	XELIRI/Bev	Lonsurf/Bev	CPT-11/Pmab	BSC		
73	f	53	T/C	Liver	WILD	12V	FOLFIRI/Cmab	Regorafenib	Lonsurf	Pmab	BSC		
74	f	71	Rectum	Lung	WILD	12S	FOLFIRI	CPT-11/Pmab	Lonsurf	BSC			
75	m	62	Rectum	Liver	WILD	12A, 12S	XELOX/Bev	XELIRI/Bev	CPT-11/Cmab	Lonsurf	BSC		
76	m	60	S/C	Liver	WILD	12C, 13D	XELOX/Bev	Lonsurf/Bev	FOLFIRI/Pmab				
77	m	72	Rectum	Liver, lung, LN	WILD	12S, 12V	XELOX/Bev	XELIRI/Bev	CPT-11/Cmab	BSC			
78	f	65	S/C	Liver, peritoneum	WILD	12D, 13D	XELOX/Bev	XELIRI/Bev	Lonsurf/Bev				
79	f	74	S/C	Liver	WILD	12D, 12S	mFOLFOX6/Pmab	FOLFIRI/Pmab	BSC				
80	m	72	A/C	Liver, LN	WILD	12V	XELOX/Bev	XELIRI/Bev	CPT-11/Cmab	Regorafenib	Lonsurf	BSC	
81	f	56	Rectum	Liver	WILD	13D	XELOX/Bev	XELIRI/Bev	BSC				
82	f	29	S/C	Peritoneum	WILD	12D	XELOX/Bev	XELIRI/Bev	CPT-11/Cmab	Lonsurf			
83	f	74	Rectum	Liver	WILD	12D	mFOLFOX6	IRIS	Pmab	Lonsurf			
84	f	50	Rectum	Liver	WILD	12A, 12C, 12D, 12V, 13D	mFOLFOX6/Bev	XELIRI/Bev	CPT-11/Cmab	Regorafenib	Lonsurf	Cmab	BSC
85	m	65	Rectum	Liver, lung	WILD	12R	XELOX/Bev	FOLFIRI/Cmab	Regorafenib	Lonsurf	CPT-11/Cmab	BSC	

A/C: ascending colon; T/C: transverse colon; D/C: descending colon; S/C: sigmoid colon; LN: lymph node; N.D.: not detected; mFOLFOX6: oxaliplatin, folinic acid, and fluorouracil; FOLFIRI: irinotecan, folinic acid, and fluorouracil; XELOX: capecitabine and oxaliplatin; XELIRI: capecitabine and irinotecan; Bev: bevacizumab; Pmab: panitumumab; Cmab: cetuximab; CPT-11: irinotecan; BSC: best supportive care.

**Table 3:** Emergence during each regimen in patients with the wild-type

	Number of patients (n)	Emergence of MctDNA (n)	Percentage of patients with emergence of MctDNA (%)^*^
Chemotherapy	4	1	25.0
Anti-VEGF antibody + Chemotherapy	33	13	39.4
Anti-EGFR antibody	25	9	36.0
Regorafenib	10	3	30.0
TAS-102	10	7	70.0

^*^Emergence of MctDNA / Number of Patients ^*^100

#### Reproducibility and sensitivity of *KRAS* monitoring

To ensure reproducibility of these dynamic changes in mutated circulating tumor DNA recognized in patients (e.g., patient 85), we conducted an additional experiment. DNA samples from patient 3 with a known *KRAS* G12C mutation were mixed with DNA samples from patient 41 at varying dilutions. DNA samples with the G12C mutation were used because no patients carried the *KRAS* G12R mutation in tumor tissues. Patient 41 tumor tissues harbored *KRAS* wild-type and showed no mutations in the blood during treatments. The number of *KRAS* G12C mutations declined as the dilution series progressed to one copy in 20000 reference copies (0.005%) and then was not detectable (data not shown). Reproducibility of the emergence of new mutations was confirmed in triplicate.

### Clinical course of two patients who showed a spike in elevation in mutated circulating tumor DNA

**Figure 5:**
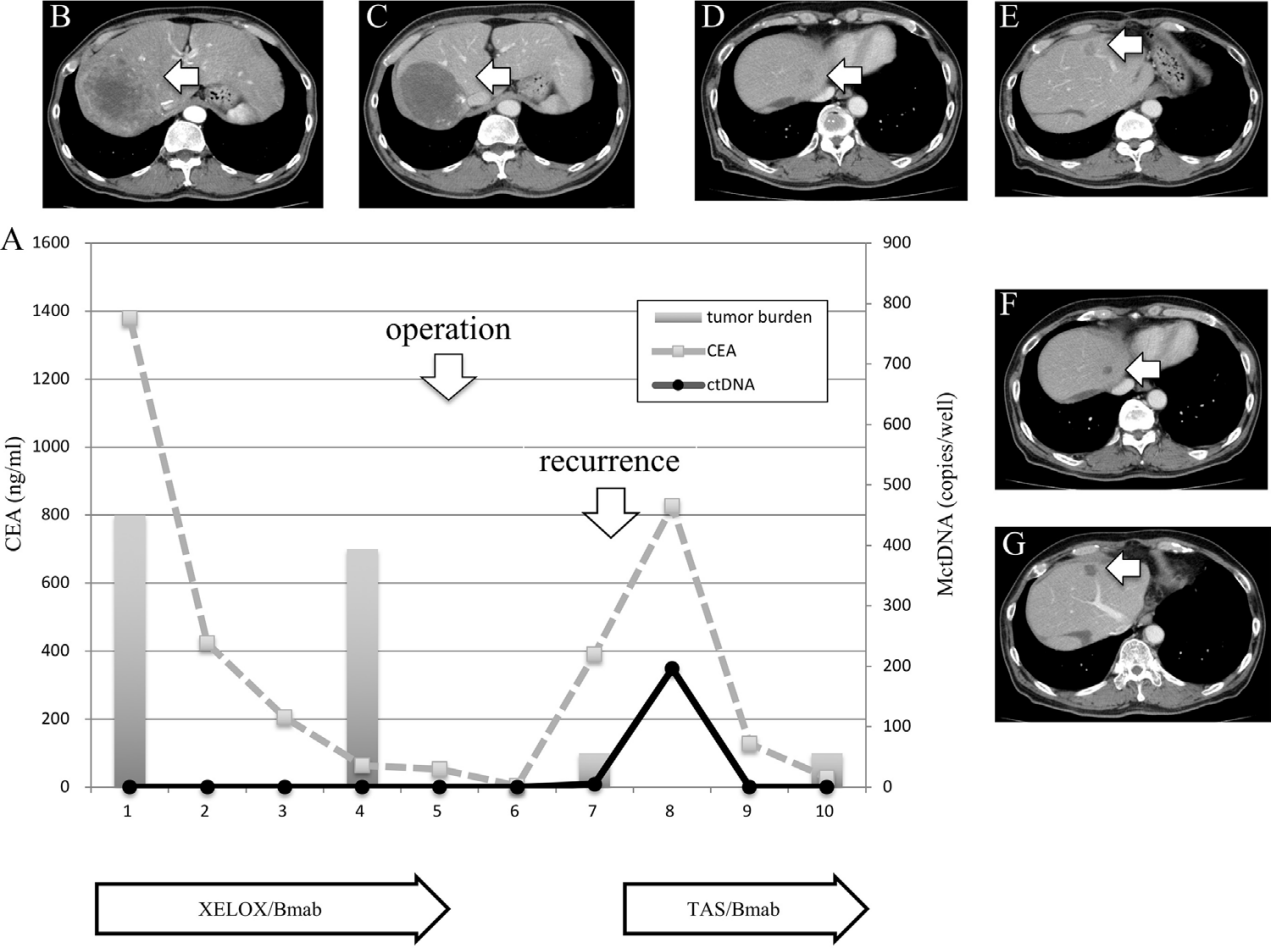
Clinical course of a mCRC patient with multiple liver metastases **(A)** with morphological changes seen with computed tomography (CT) **(B–G)** and a spike in elevation in MctDNA. A 60-year-old male with multiple liver metastases is denoted as patient 76. He was treated with XELOX + bevacizumab as the first-line treatment. (B) and (C) show CT images before and after treatment with XELOX + bevacizumab. A change in tumor morphology from heterogeneous to homogeneous low-attenuation was seen in the liver metastases four cycles after treatment with XELOX + bevacizumab despite no change in tumor size. The patient found XELOX + bevacizumab treatment difficult because of severe adverse events and he underwent surgery (right lobectomy for the main tumor and partial resection for other multiple metastases). Soon after surgery, a recurrent liver tumor was found along with increased levels of carcinoembryonic antigen (CEA). Chemotherapy was suggested but the patient refused because of the previous severe adverse events with the first-line treatment. TAS-102 + bevacizumab was then suggested because TAS-102 did not show severe adverse events. Soon after treatment with TAS-102 + bevacizumab, CEA drastically decreased and liver tumors showed morphological changes, which were also seen with the first-line treatment. Additionally, a spike in elevation in MctDNA was observed during this drug response.

**Figure 6:**
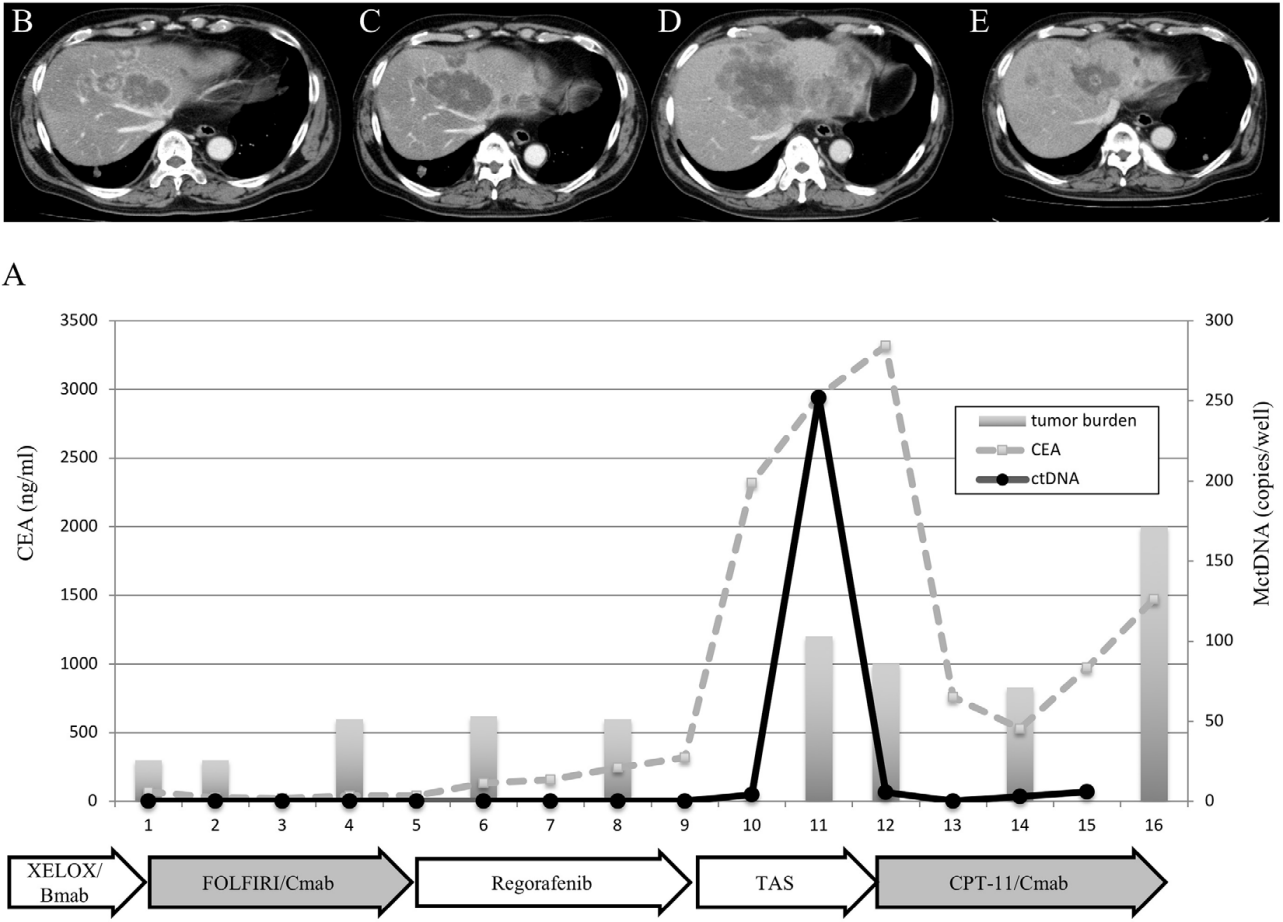
Clinical course of a mCRC patient with multiple liver metastases **(A)** treated with reintroduction of CPT + cetuximab because of the disappearance of MctDNA. A 65-year-old male with multiple liver metastases is denoted as patient 85. He was treated with XELOX + bevacizumab as the first-line treatment, FOLFIRI + cetuximab as the second-line treatment, regorafenib as the third-line treatment, and TAS-102 as the fourth-line treatment.

A spike in elevation was seen in six patients treated with anti-VEGF antibody or TAS-102. Although detection of MctDNA was generally seen in patients with disease progression, a spike in elevation of MctDNA was observed in patients who likely responded to drug treatments, followed by a quick disappearance. A patient who showed a drug response with a spike in elevation is shown in Figure 5. Despite there being no change in tumor size, tumors did show changes in morphology induced by TAS-102 + bevacizumab as the second-line treatment. It is reported that patients with a change in morphology show as good a drug response as those patients with a change in size, similar to a partial response and a complete response, estimated using Response Evaluation Criteria in Solid Tumours (RECIST) in not only colorectal cancer but other types of tumors such as gastrointestinal stromal tumors [23, 24, 25]. Interestingly, this patient also showed a change in tumor morphology with first-line treatment using XELOX + bevacizumab. There were some patients with a decline in MctDNA, suggesting recovery of drug sensitivity, who were then treated with re-introduction of anti-EGFR antibody. Figure 6 shows a representative image of a patient who responded to re-introduction of anti-EGFR antibody. The patient achieved a partial response and 7 months PFS with the sixth-line treatment.

### Comparison of KRAS mutations between blood and tumor tissues

In tumors of patients with WT *KRAS* who showed *KRAS* mutations in blood, the presence or absence of identical mutations were investigated in primary tumors using droplet digital PCR (ddPCR). Table 4 shows a comparison of point mutations in *KRAS* codon 12/13 between blood and tumor tissues in WT patients. Mutations shared between blood and tumor tissues were seen in 11 patients (73.3%). We also confirmed the accuracy of ddPCR by exploring each mutation using matched normal colorectal tissues from 33 CRC patients as negative controls. No mutations, except G12C and G12D, were detected in matched normal colorectal tissues of all 33 CRC patients. G12C was detected in one patient (3.0%) and G12D was detected in two (6.1%). These results indicated that some cases could be false positive by appearing to harbor G12C or G12D mutations; therefore, results should be interpreted with caution. In cases where G12C or G12D mutations are detected, this should be reevaluated using other samples collected on a different day.

**Table 4:** Comparison between blood and primary tissues for mutations in 15 patients

No.	Mutation in blood	Mutation in primary tissues
58	12S	12S
59	12V	N.D.
62	12S	12S
64	12R	12D, 12S, 13D
65	12C, 13D	12S, 13D
68	12S	12S
69	13D	12S, 13D
75	12A, 12S	12S
76	12C, 13D	13D
77	12S, 12V	N.D.
78	12D, 13D	12D, 13D
81	13D	12D, 12S, 13D
83	12D	12D
84	12A, 12C, 12D, 12V, 13D	12D
85	12R	12S, 13D

N.D.: not detected.

### Correlation between mutated circulating tumor DNA and carcinoembryonic antigen

To clarify the characteristics of MctDNA, we examined correlations between MctDNA and carcinoembryonic antigen, a conventional tumor marker used to assess disease progression. The correlation for MT was stronger than that for WT for both number (r_s_ = 0.53, p < 0.01 and r_s_ = 0.33, p < 0.01, respectively) and ratio (r_s_ = 0.50, p < 0.01 and r_s_ = 0.31, p < 0.01, respectively).

## DISCUSSION

We investigated dynamic changes in MctDNA during various regimens to provide useful information for the treatment of patients with mCRC.

MctDNA was seen in the blood of patients with not only the MT but also the WT in tumor tissues. MctDNA was observed during treatments with various drugs such as anti-VEGF antibody, regorafenib, TAS-102, and anti-EGFR antibody. Regardless of the *KRAS* status in tumor tissues, patients with MctDNA in blood showed poor PFS with first-line treatment. *KRAS* monitoring identified dynamic changes in MctDNA, such as continuous, intermittent, and transient changes, which corresponded with drug response or resistance. It is possible that the disappearance of MctDNA could be involved in recovery of sensitivity to anti-EGFR antibody [19].

MctDNA number and ratio were compared between MT and WT patients. Median values for MT patients were 64.0 copies/well for the number and 10.10% for the ratio, and were 9.1 copies/well for the number and 0.22% for the ratio for WT patients (Figure 1B and 1C). WT patients showed 1/7 the level of MctDNA compared with MT patients (64.0 *vs.* 9.1), suggesting that WT patients had *KRAS* mutant cells in 1/7 (14.7%) of tumors. Some smaller pieces of these mutant cells may reach the blood, resulted in the low ratio of *KRAS* mutant cells (0.22%) in blood of WT patients. The lowest ratio (0.002%) among total cell-free DNA was detected using ddPCR in this analysis. WT patients showed fluctuating changes in the ratio of MctDNA, at around 0.01% of the detection sensitivity of ddPCR, which may have resulted in intermittent detection of MctDNA in WT patients. In contrast, MT patients showed a high ratio of MctDNA (10.10% (0.26–93.60)), allowing for the continuous detection of MctDNA. The lowest ratio (0.26%) was well within the detection range for ddPCR, with a detection sensitivity of 0.01%.

MctDNA was observed in blood of WT patients during treatment with different drugs, such as anti-VEGF antibody, regorafenib, TAS-102, and anti-EGFR antibody. Studies have reported that anti-EGFR antibody is likely involved in the emergence of ctDNA. A recent clinical trial reported ctDNA in 20% of patients treated with anti-VEGF antibody in subgroup analysis [26]. This trial was a randomized phase II study to assess FOLFIRI + bevacizumab beyond progression and FOLFIRI + panitumumab as a second-line treatment for patients with *KRAS* WT mCRC. No significant differences in PFS were observed in patients without the emergence of ctDNA. Twenty percent of patients with ctDNA showed an extremely poor outcome with the second-line treatment when treated with anti-EGFR antibody. The problem is that these patients are good candidates for anti-EGFR antibody treatment because they have no *KRAS* mutations in tumor tissues before treatment. The trial indicated that 20% of patients are unlikely to respond to anti-EGFR antibody after prior administration of anti-VEGF antibody. An altered *KRAS* status is implicated in the sub-sequential treatment outcome; therefore, *KRAS* monitoring is essential for the treatment of mCRC patients to provide appropriate drug strategies.

There are two possible factors that may contribute to the mechanisms underlying the emergence of *KRAS* mutations in the blood. First, an acquired *KRAS* mutation in the tumor may travel to the blood. Anti-VEGF antibody is directed against the tumor vasculature, and should destroy the tumor vasculature, thereby depriving the tumor of oxygen and nutrients [27, 28, 29]. Glucose deprivation in tumors was reported to induce *KRAS* mutations [30], suggesting insufficient oxygen or nutrients in the tumor induced by anti-VEGF antibody may be involved in this mechanism [27, 28, 29]. Regorafenib is a molecular target drug aimed at inhibiting the VEGF signaling pathway. Anti-EGFR antibody is also reported to induce acquired mutations *in vitro*, but the mechanism is not well understood [10]. Additionally, tumors in WT patients with latent mutant cells, undetectable by conventional PCR methods with a sensitivity of 1% [31, 32, 33], may expand as a consequence of the treatment, becoming detectable in blood. In the current study, ddPCR with a high sensitivity was able to detect latent mutant cells in tumor tissues. Our data revealed mutations that were shared by both tumor tissues and blood, suggesting that tumor cells with acquired *KRAS* mutations may travel to the blood. Different types of *KRAS* mutations were also observed suggesting that latent cells from the tumors, with undetected *KRAS* mutations may undergo clonal expansion during treatment. The possibility that de novo mutations may arise from within some of the various types of blood cells seems unlikely.

*KRAS* monitoring identified continuous, intermittent, and transient changes in MctDNA. Continuous detection of MctDNA was frequently seen in MT patients, whereas intermittent detection was more often seen in WT patients. These changes may be associated with the different levels of MctDNA observed between WT and MT patients. In WT patients treated with anti-EGFR antibody, initial detection of MctDNA was likely prior to radiological disease progression (Figure 1D) [10, 9, 19]. Although detection of MctDNA was generally seen in patients with disease progression, transient changes with a spike in elevation were seen in patients in association with the drug response (Figures 3H and 5). One patient showed a transient change with a spike in elevation during treatment with TAS-102, followed by disease progression (Figure 6). MctDNA then disappeared in this patient and anti-EGFR antibody was reintroduced, which achieved a partial response and a long PFS of 7 months with the fifth-line treatment. The results suggest that the later treatment lines had a significant effect on improving the outcome for this patient. The rapid disappearance may have been induced by a delayed drug response to TAS-102 [34]. TAS-102 has a unique mechanism and works by being integrated into the DNA of the tumor cells. Such a process requires some time before an effect on the tumor is observed [35, 36]. The spike in elevation followed by disease progression may indicate a delayed drug response of TAS-102 and contributing effects associated with anti-EGFR antibody.

In conclusion, although our results should be interpreted within the study limitations and further examinations are required to draw a definitive conclusion, *KRAS* monitoring seems to be a useful tool to help determine treatment strategies. The dynamics of ctDNA during *KRAS* monitoring provide important information that may aid the treatment of mCRC patients.

## MATERIALS AND METHODS

### Patients and study design

We prospectively recruited 85 patients (47 males and 38 females) with histologically confirmed mCRC with distant metastases and collected 457 blood samples between June 2014 and March 2017 at the Saitama Medical Center, Jichi Medical University, Japan. Patients were aged >18 years, and their Eastern Cooperative Oncology Group performance status was 0, 1, or 2. Disease extension and response were assessed using computed tomography and the clinical response was evaluated according to RECIST 1.1 criteria. The characteristics of the 85 patients are shown in Table 5.

**Table 5:** Patient characteristics

*KRAS* codon 12/13 mutation in primary tumor		No mutation (wild-type) (n = 56)	Mutation (n = 29)	p-value
Gender				0.663
	Male	30	17	
	Female	26	12	
Mean age (range)		64.1 (29–79)	65.7 (19–82)	0.219
Location (primary CRC)				0.278
	Right	15	13	
	Left	25	8	
	Rectum	16	8	
Treatment line				0.825
	1st line	29	14	
	2nd line	13	10	
	3rd line	8	3	
	4th line	5	1	
	BSC	1	1	
Treatment change				0.479
	0	33	13	
	1	16	15	
	2	4	1	
	3	3	0	
CEA at initial assessment	Median (range)	10.7 (0–1379)	24.6 (1.8–3110)	0.262
Blood collection	Median (range)	4 (1–16)	4 (1–12)	0.196
Follow-up months	Median (range)	16.5 (3–34)	12 (3 - 28)	0.192
Differentiation				0.975
	Pap+well+mod	52	27	
	Muc+por+sig	4	2	
Primary tumor				0.4
	Not resected	12	4	
	Resected	44	25	
Solitary/synchronous				0.205
	Solitary	31	20	
	Synchronous	25	9	
Metastatic organ				0.2
	1	31	14	
	2	22	10	
	3	2	5	
	0	1	0	

BSC: best supportive care; CRC: colorectal cancer; CEA: carcinoembryonic antigen; pap: papillary; mod: moderate; muc: mucinous; sig: signet ring.

The study was approved by the Research Ethics Committee at Jichi Medical University and was conducted in accordance with the principles contained within the Declaration of Helsinki. Written informed consent was obtained from the study participants.

### Analysis of KRAS status in primary tumor tissues

*KRAS* status was evaluated using the Scorpion amplified refractory mutation system method or a RASKET kit using formalin-fixed paraffin-embedded tumor tissues from patients. *KRAS* analysis was performed by a clinical testing company (Special Reference Laboratories, Tokyo, Japan).

### Plasma sample collection and extraction of circulating cell-free DNA

Blood samples were processed for plasma within 5 h of collection. Blood (7 mL) was taken from each patient, and plasma was collected by centrifugation at 3000 × *g* for 20 min at 4°C, followed by centrifugation at 16000 × *g* for 10 min at 4°C in a fresh tube. The supernatant was immediately collected and stored at −80°C until DNA extraction. Circulating cell-free DNA was extracted from 2 mL of plasma using a QIAamp circulating nucleic acid kit (Qiagen, Tokyo, Japan) according to the manufacturer's instructions.

### Droplet digital PCR analyses

The *KRAS* status in ctDNA was determined using ddPCR (Bio-Rad, Tokyo, Japan). Seven *KRAS* mutations (G12D, G12V, G12C, G12R, G12A, G12S, and G13D) were assessed. The ddPCR mixture contained 10 μL of 2 × ddPC Supermix, 250 nM of forward and reverse primers, 62.5 nM MT and WT probe, and 8 μL of sample eluted from plasma. The reaction mixture (20 μL) was loaded into a DG8 cartridge (Bio-Rad) with a gasket and the cartridge was placed into the droplet generator, according to the manufacturer's instructions. The generated droplets were transferred into a 96-well plate and sealed using a foil lid and a thermal plate sealer. After heat sealing, PCR was performed using a Veriti thermal cycler (Thermo Fisher Scientific, Waltham, MA, USA) under the following conditions: 10 min at 95°C, 39 cycles at 94°C for 30 s, then at 60°C for 60 s. Amplified droplets were analyzed using a QX200 droplet reader (Bio-Rad) for fluorescent measurement of FAM probes for WT and HEX for MT. ddPCR data were analyzed using QuantaSoft software. Amplified DNA products were extracted from droplets following PCR for Sanger sequencing. Samples with two or more positive droplets were determined as positive. To explore the reproducibility and sensitivity of the methods, for instance, a tumor with a known mutation that is also found in the plasma could be spiked into DNA samples from other cases to see if the mutation could be detected at varying dilutions.

### Threshold values for droplet digital PCR

To determine the number of positive droplets required for a true positive for MctDNA, we confirmed the sequence of the mutation according to the number of droplets obtained. Different numbers of droplets (1, 2, 4, and 5) were sorted using the On-chip Sort system (On-chip Biotechnologies, Tokyo, Japan) and the sequence of the mutation was confirmed using the HCT-116 cell line, which has a *KRAS* codon 13 mutation. Clinical samples with the *KRAS* mutation were used to verify the sequence of the mutation. Only one positive droplet obtained by sorting showed a negative result in one out of three examinations (33.3%); whereas two or more positive droplets did not fail to show the mutation in three examinations. Samples with two or more positive droplets were determined as being positive.

### Sorting positive droplets using the On-chip Sort system

To verify the sequence of PCR fragments in droplets, the On-chip Sort system was used to sort positive droplets that were labelled with FAM, according to the manufacturer's instructions. The instrument was a microfluidic chip-based cell sorter that allows for the use of any liquid as a carrier fluid. Selected FAM-positive droplets were confirmed by fluorescent microscopy and DNA fragments were collected from droplets by extraction with chloroform.

### TA cloning and Sanger sequencing

Collected DNA fragments were amplified using the prime PCR for ddPCR *KRAS* assay (Bio-Rad), and then used for TA cloning after elimination of DNA fragments with the WT. PCR products were used with the TOPO TA cloning kit for Sanger sequencing (Invitrogen, Carlsbad, CA, USA), according to the manufacturer's instructions. Plasmid DNA was extracted using a QIAprep spin miniprep kit (Qiagen) and Sanger sequencing was performed using an ABI 3130xl genetic analyzer (Applied Biosystems, Foster City, CA, USA).

### Statistical analysis

Fisher's exact test was used to examine the relationship between two categorical variables. Comparison of continuous variables between two groups was performed, with Student's *t*-test being used for those variables with a normal distribution and the non-parametric Mann–Whitney–Wilcoxon test being used for those variables without a normal distribution. The association between ctDNA and carcinoembryonic antigen was determined using Spearman's correlation test. A p-value of 0.05 was considered statistically significant. All analyses were conducted using StatView ver. 5.0 (SAS Institute, Inc., Cary, NC, USA).

## Abbreviations

ctDNA: circulating tumor DNA
ddPCR: droplet digital PCR
EGFR: epidermal growth factor receptor
mCRC: metastatic colorectal cancer
MctDNA: *KRAS* mutated circulating tumor DNA
MT: mutant-type
PFS: progression-free survival
VEGF: vascular endothelial growth factor
WT: wild-type
